# Mechanism of Inducible Nitric Oxide Synthase Exclusion from Mycobacterial Phagosomes

**DOI:** 10.1371/journal.ppat.0030186

**Published:** 2007-12-07

**Authors:** Alexander S Davis, Isabelle Vergne, Sharon S Master, George B Kyei, Jennifer Chua, Vojo Deretic

**Affiliations:** Department of Molecular Genetics and Microbiology, University of New Mexico School of Medicine, Albuquerque, New Mexico, United States of America; Johns Hopkins School of Medicine, United States of America

## Abstract

Mycobacterium tuberculosis is sensitive to nitric oxide generated by inducible nitric oxide synthase (iNOS). Consequently, to ensure its survival in macrophages, M. tuberculosis inhibits iNOS recruitment to its phagosome by an unknown mechanism. Here we report the mechanism underlying this process, whereby mycobacteria affect the scaffolding protein EBP50, which normally binds to iNOS and links it to the actin cytoskeleton. Phagosomes harboring live mycobacteria showed reduced capacity to retain EBP50, consistent with lower iNOS recruitment. EBP50 was found on purified phagosomes, and its expression increased upon macrophage activation, paralleling expression changes seen with iNOS. Overexpression of EBP50 increased while EBP50 knockdown decreased iNOS recruitment to phagosomes. Knockdown of EBP50 enhanced mycobacterial survival in activated macrophages. We tested another actin organizer, coronin-1, implicated in mycobacterium-macrophage interaction for contribution to iNOS exclusion. A knockdown of coronin-1 resulted in increased iNOS recruitment to model latex bead phagosomes but did not increase iNOS recruitment to phagosomes with live mycobacteria and did not affect mycobacterial survival. Our findings are consistent with a model for the block in iNOS association with mycobacterial phagosomes as a mechanism dependent primarly on reduced EBP50 recruitment.

## Author Summary

Mycobacterium tuberculosis infects one third of the world's population, with the majority of infected individuals being asymptomatic while running a lifetime risk of developing active disease. The key to the success of M. tuberculosis as a recalcitrant human pathogen is its ability to parasitize macrophages and persist in these cells or their derivatives for long periods of time. We still do not have complete knowledge of the full repertoire of M. tuberculosis determinants that allow it to evade bactericidal mechanisms of the macrophage. Here we report the mechanism by which M. tuberculosis eludes effective elimination by nitric oxide, a radical with antimycobacterial properties that is generated by the inducible form of nitric oxide synthase. It was generally assumed that nitric oxide synthase, upon induction by the major anti-tuberculosis cytokine interferon gamma, simply homogeneously fills up the macrophage like a sack and generates nitric oxide throughout the cell. The present study shows that nitric oxide synthase is not randomly distributed in macrophages, and that its positioning in the cell is dictated by interactions with the scaffolding protein EBP50, shown here to be induced during macrophage activation. Thus, not only do the phagocytic cells increase the amount of nitric oxide synthase, but they also have a system to deliver and keep this enzyme in the vicinity of phagosomes. This is of significance, as nitric oxide is a highly reactive radical, and its generation somewhere else in the cell would lead to it being spent by the time it diffuses to the site of intended action, such as mycobacterium-laden phagosomes. It turns out, as this study shows, that M. tuberculosis interferes with the process of EBP50-guided positioning of the inducible nitric oxide synthase, thus avoiding delivery and accumulation of this enzyme and its noxious products near the phagosome where nitric oxide would have the best chance of inhibiting intracellular mycobacteria.

## Introduction

Approximately one-third of the world's population is asymptomatically infected with *Mycobacterium tuberculosis.* This represents the reservoir leading to 8 million cases of active disease and 2 million deaths annually from tuberculosis. Being an intracellular pathogen, M. tuberculosis is able to infect and replicate in host macrophages by defending against and manipulating macrophage antimicrobial responses [1]. After entry into macrophages via phagocytosis, M. tuberculosis has the capacity to avoid phagosome-lysosome fusion by interfering with phagolysosome biogenesis pathways, a trait that has been well recognized and studied [2–6]. The important questions that have not been fully addressed thus far are whether or not and how M. tuberculosis manipulates other macrophage microbicidal responses aside from phagolysosomal biogenesis, such as the production of reactive oxygen intermediates and reactive nitrogen intermediates (RNI).

The production of nitric oxide (NO**^.^**) and other reactive nitrogen metabolites has been shown to play an important effector role in innate immunity [7]. NO**^.^** is a highly reactive and diffusible free radical, soluble in both lipids and water, and capable of reacting with oxygen and reactive oxygen intermediates to form NO_2_, NO_2_^−^, NO_3_^−^, N_2_O_3_, and the highly mycobactericidal ONOO^−^ [8–10]. Inducible nitric oxide synthase (iNOS) is the major generator of RNI in immune cells and is a tightly regulated enzyme [11]. It has been demonstrated that iNOS is important in immune control of M. tuberculosis as evidenced by compromised handling of infections in mice lacking the gene for iNOS [12]; iNOS is believed to perform a similar function in human macrophages, although this issue has not been fully resolved [12]. Studies investigating defenses against RNI in M. tuberculosis have demonstrated the importance of several mycobacterial proteins against RNI [13,14], further underscoring the potential importance of iNOS in host protection against M. tuberculosis. Since iNOS products are highly reactive radicals, if NO**^.^** is generated at intracellular sites distant from intended targets (e.g., away from phagosomes containing bacteria), it may be consumed or spent before it can reach the microbe. Thus, the proximity of iNOS to the intracellular targets is likely to play a role. Recently, it was reported that M. tuberculosis inhibits trafficking of iNOS to the mycobacterial phagosome [15].

Here, we investigated the mechanism by which iNOS is normally recruited to phagosomes and the properties of the mycobacterial phagosome responsible for iNOS exclusion. Since trafficking of iNOS to phagosomes in a macrophage depends on a functional actin cytoskeleton [15], we looked at actin cytoskeleton regulation and iNOS scaffolding molecules interacting directly or indirectly with actin. Ezrin/radixin/moesin (ERM)-binding phosphoprotein 50 (EBP50), also known as Na/H+ exchange regulatory factor 1, is a scaffolding protein responsible for the anchoring of various cellular proteins to the actin cytoskeleton through its linkage to ERM proteins. A 358-residue protein, with an ERM-binding domain and two PSD-95/Dlg-1, *Drosophila* disk large/ZO-1 (PDZ) domains, EBP50 has been shown to bind to iNOS through one of its PDZ domains and the SAL motif at the C-terminal end of iNOS [16]. We report that the localization of iNOS to phagosomes is an EBP50-dependent process and that EBP50, like iNOS, is upregulated in macrophages activated with interferon-gamma (IFN-γ) and lipopolysaccharide (LPS). EBP50 is targeted by live mycobacteria to inhibit recruitment of iNOS to the vicinity of their phagosomes. This provides a new understanding of how iNOS is trafficked in macrophages.

## Results

### Inhibition of iNOS Recruitment to the Phagosome Requires Mycobacterial Viability

**Figure 1:**
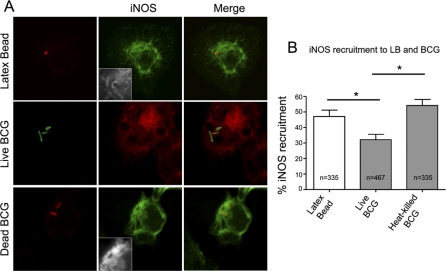
Live Mycobacteria Disrupt Proper Localization of iNOS to the Phagosome Macrophages were stimulated with IFN-γ and LPS for 16 h and infected with Texas Red–labeled latex beads or M. tuberculosis var. *bovis* BCG for 10 min. After 1-h chase, cells were fixed, stained for iNOS, and examined by confocal microscopy. (A) Localization of iNOS (green/red) to the phagosome is observed for both latex bead (red) and dead mycobacterial phagosomes (red), but missing from live mycobacterial phagosomes (green). (B) Quantification, using previously reported methods [15] of iNOS localized to phagosomes in cells infected with latex beads, live, or dead mycobacteria. Bars, mean ± s.e.m. (three independent experiments); *n*, number of phagosomes counted per condition. ^*^ *p* = 0.0484 (latex bead versus live), 0.0142 (live versus dead).

Model latex bead phagosomes have been used to demonstrate actin-dependent recruitment of iNOS to the vicinity of phagosomes formed in IFN-γ- and LPS-activated macrophages [15]. This process is disrupted when cells are infected with M. tuberculosis. Since mycobacterial factors affecting intracellular trafficking in infected cells include preformed products such as lipids [6], we tested whether mycobacterial viability was required for iNOS exclusion. To induce iNOS expression, RAW 264.7 macrophages were treated with IFN-γ and LPS for 16 h, infected for 10 min with latex beads, live or heat-killed M. tuberculosis var. *bovis* BCG labeled with Texas Red under conditions not affecting bacterial viability [4], and localization of endogenous iNOS relative to the phagosomes analyzed by fluorescence confocal microscopy (Figure 1A). In accordance with previous studies, phagosomes harboring live mycobacteria showed a reduction in iNOS recruitment as compared to latex bead phagosomes (32% ± 3% versus 47% ± 4%, *p* = 0.0484) (Figure 1B). In contrast, heat- killed mycobacteria were unable to disrupt the localization of iNOS to the phagosome when compared to latex bead phagosomes (54% ± 4% versus 47% ± 4%, *p* = 0.2870) (Figure 1B). These results indicate that the inhibition of iNOS recruitment to the vicinity of mycobacterial phagosomes is an active mechanism that requires live mycobacteria.

### EBP50 Is Recruited to Phagosomes and Colocalizes with iNOS in Macrophages

**Figure 2:**
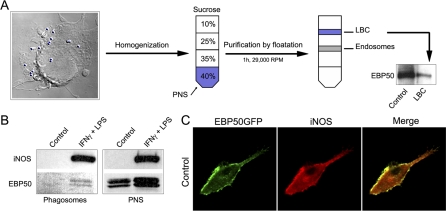
EBP50 Copurifies with Latex Bead Phagosomes and Colocalizes with iNOS (A) Latex bead phagosome preparation according to the previously published procedures [27]. Macrophages were infected with latex beads for 1 h, homogenized, and latex bead compartments isolated by floatation in a sucrose gradient. Western blot of 50 μg of protein per lane developed with EBP50 antibody. PNS, post-nuclear supernatant. Control, PNS; LBC, latex bead compartment. (B) iNOS and EBP50 are present on isolated magnetic bead phagosomes. RAW 264.7 macrophages were untreated (control) or activated for 16 h with IFN-γ and LPS prior to phagocytosis. Magnetic beads were phagocytosed for 1 h, and post-nuclear-supernatant and phagosomes were prepared and analyzed by western blot. (C) Macrophages were transfected with EBP50-GFP (green) and stimulated with IFN-γ and LPS for 16 h. Cells were fixed, stained for endogenous iNOS (red), and examined by confocal microscopy.

In polarized epithelial cells, iNOS localization depends on EBP50 [16]. Here we tested the role of EBP50 on iNOS distribution in macrophages. Latex bead phagosomes were isolated as previously described [15] (Figure 2A) and probed for EBP50 by western blotting (Figure 2A). Endogenous EBP50 was detected on latex bead compartments, purified by floatation. This was confirmed using an independent phagosome purification protocol, with phagosomes containing magnetic beads. Along with EBP50, iNOS was present on purified model magnetic bead phagosomes (Figure 2B). This result indicates that iNOS and EBP50 are both recruited to model latex bead, or magnetic bead phagosomes. Furthermore, EBP50-GFP colocalized with iNOS in macrophages (Figure 2C).

### EBP50 Controls Recruitment of iNOS to Phagosomes

**Figure 3:**
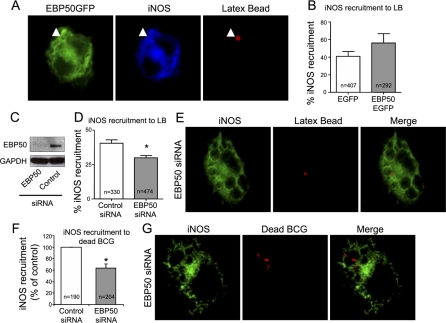
EBP50 Controls iNOS Localization Relative to Phagosomes (A and B) Macrophages were transfected with EBP50-GFP, stimulated with IFN-γ and LPS for 16 h, and then allowed to phagocytose Texas Red–labeled latex beads for 10 min. After 1-h chase, cells were fixed, stained for iNOS, and examined by confocal microscopy. (A) Confocal microscopy images showing localization (triangle) of EBP50-GFP (green) and iNOS (blue) to latex bead phagosomes (red). (B) Quantification of iNOS localization to latex bead phagosomes. Bars, mean ± s.e.m. (three independent experiments); *n*, number of phagosomes counted per condition. (C–E) Macrophages were transfected with either control (scramble) or EBP50 siRNA, stimulated with IFN-γ and LPS for 16 h, and then allowed to phagocytose Texas Red–labeled latex beads for 10 min. After 1-h chase, cells were fixed, stained for iNOS, and examined by confocal microscopy. (C) Western blot of EBP50 and loading control (GAPDH) after 48 h (includes IFN-γ and LPS stimulation). (D) Quantification of iNOS recruitment to latex bead phagosomes. Bars, mean ± s.e.m. (three independent experiments); *n*, number of phagosomes counted per condition. ^*^ *p* = 0.0254. (E) Absence of iNOS localization (green) to latex bead phagosomes (red). (F and G) Macrophages were transfected with either control (scramble) or EBP50 siRNA, stimulated with IFN-γ and LPS for 16 h, and then allowed to phagocytose Alexa 568–labeled dead mycobacteria for 10 min. After 1-h chase, cells were fixed, stained for iNOS, and examined by confocal microscopy. (F) Quantification of iNOS recruitment to dead mycobacteria phagosomes. The results are represented as percent relative to control siRNA. Bars, mean ± s.e.m. (three independent experiments); *n*, number of phagosomes counted per condition. *p = 0.049. (G) Absence of iNOS localization (green) to dead mycobacteria phagosomes (red).

A functional role of EBP50 in iNOS recruitment to phagosomes was tested next. Activated macrophages expressing EBP50-GFP were infected with latex beads and analyzed by fluorescence microscopy for localization of endogenous iNOS to model latex bead phagosomes. Latex bead phagosomes in cells expressing EBP50-GFP showed some increase in iNOS recruitment to the latex bead phagosomes when compared to cells expressing control enhanced green fluorescent protein (56% ± 11% versus 41% ± 5.5%), albeit the statistical significance cut-off of <0.05 was not reached (Figure 3A and 3B). Nevertheless, this trend suggested that EBP50 might play a role in trafficking of iNOS to phagosomes. To test this, short interfering RNA (siRNA) directed against EBP50 was used to knockdown the endogenous EBP50. Macrophages were transfected with either control (scramble) siRNA or EBP50 siRNA, treated with IFN-γ and LPS for 16 h, and then infected with latex beads. EBP50 knockdown expression was confirmed by western blotting (Figure 3C). In cells with EBP50 siRNA, there was a decrease in recruitment of iNOS to the latex bead phagosome as compared to cells transfected with control siRNA (30% ± 2% versus 40% ± 2.5% *p* = 0.0254) (Figure 3D and 3E). This level of decrease was similar to that observed in comparisons between phagosomes harboring live mycobacteria, dead mycobacteria, or latex beads (Figure 1). These results indicate that EBP50 plays a role in the recruitment of iNOS to latex bead phagosomes. Finally, we tested the involvement of EBP50 in recruitment of iNOS to mycobacterial phagosomes. In cells transfected with EBP50 siRNA, iNOS localization to phagosomes containing dead mycobacteria was reduced by 37% ± 7% compared to cells transfected with control siRNA (Figure 3F and 3G). Overall, these results demonstrate that EBP50 is essential for recruitment of iNOS to model and mycobacterial phagosomes.

### EBP50 Expression Is Increased upon IFN-γ and LPS Stimulation

**Figure 4:**
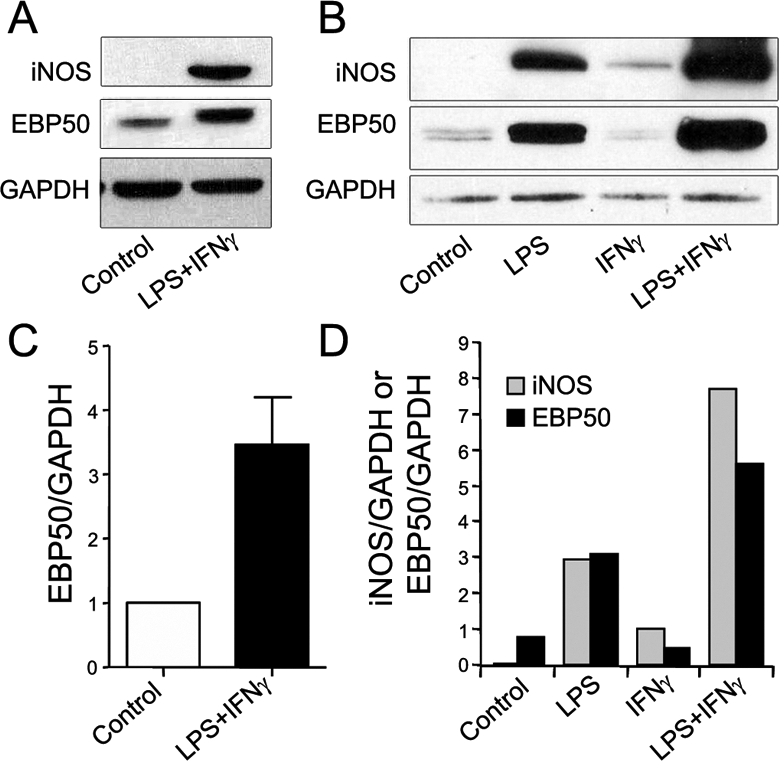
EBP50 Expression Is Increased upon IFN-γ and LPS Stimulation Macrophages were stimulated with IFN-γ and LPS or left in media for 16 h, lysed, and analyzed by western blot. (A) Expression of iNOS, EBP50, and loading control (GAPDH) in either media or media with IFN-γ and LPS. (B) Expression of iNOS, EBP50, and loading control (GAPDH) in untreated (control), LPS-treated, IFN-γ-treated, or LPS- + IFN-γ-treated cells. (C) Quantification of increase in EBP50 expression. Bars, mean ± s.e.m (three independent experiments). (D) Quantification of EBP50/GAPDH and iNOS/GAPDH in untreated (control), LPS-treated, IFN-γ-treated, or LPS + IFN-γ-treated cells.

Expression of iNOS is induced upon macrophage stimulation with IFN-γ and LPS. If EBP50 controls iNOS localization, we hypothesized that EBP50 expression might also be affected. We tested whether EBP50 protein levels changed upon macrophage activation with IFN-γ and LPS. Cells were incubated in media (control) or media containing IFN-γ (200 U/ml) and LPS (200 ng/ml) for 16 h, and then protein extracts analyzed for EBP50 content by western blotting. As expected, iNOS was undetectable under resting conditions, but was strongly upregulated upon treatment of IFN-γ and LPS (Figure 4A). EBP50 showed a similar induction pattern, although some EBP50 was detectable even without activation. Upon stimulation, there was a strong increase in EBP50 levels (Figure 4A and 4C). Increase in expression of EBP50 by IFN-γ and LPS parallels that of iNOS, consistent with a model in which EBP50 directs the proper trafficking of iNOS in macrophages. To determine the contribution of different signaling pathways (LPS and IFN-γ) in EBP50 induction, macrophages were treated separately with LPS or IFN-γ. EBP50 was upregulated upon treatment with LPS but not with IFN-γ alone; iNOS was also upregulated with LPS and to a lesser extent with IFN-γ alone (Figure 4B and 4D). The expression of EBP50 was further increased when IFN-γ was included along with LPS treatment (Figure 4B and 4D). These data indicate that EBP50 expression is regulated primarily by the LPS signaling pathway, and that, as for iNOS, IFN-γ synergizes with the LPS effect.

### Live Mycobacteria Release EBP50 Rapidly from Phagosomal Membrane

**Figure 5:**
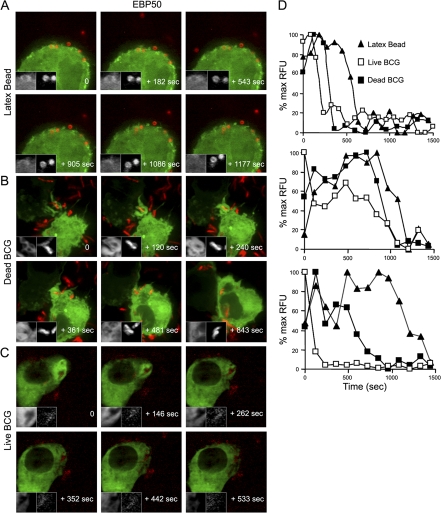
Faster Desorption of EBP50-GFP from Phagosomes Containing Live Mycobacteria Macrophages were transfected with EBP50-GFP and stimulated with IFN-γ and LPS for 16 h. Cells were infected with Texas Red–labeled latex beads, live or dead mycobacteria, and analyzed by live confocal microscopy. (A–C) Frames from time-lapse microscopy illustrate that phagosomes with latex beads (A) and dead mycobacteria (B) acquire and retain EBP50 longer than live mycobacteria (C) which rapidly lose EBP50. (D) Quantification of EBP50-GFP recruitment to phagosomes. Percent phagosome fluorescence relative to the maximum fluorescence intensity is shown as a function of time. Kinetics of three independent experiments for latex bead, dead mycobacteria, and live mycobacteria phagosomes are represented.

To investigate whether EBP50 played a role in mycobacterial effects on iNOS distribution, we examined recruitment of EBP50 to the phagosomes containing latex beads, live and dead mycobacteria, applying the previously described 4-D confocal microscopy approach [17]. Macrophages were transfected with EBP50-GFP, treated with IFN-γ and LPS for 16 h, and then allowed to phagocytose latex beads or mycobacteria. The dynamics of EBP50 recruitment to the phagosome was monitored by live confocal microscopy. Latex bead phagosomes rapidly acquired EBP50-GFP upon entry into macrophages (Figure 5A and 5D, Video S1), followed by its loss at later time points. A rapid recruitment of EBP50 was also observed with dead and live mycobacteria phagosomes (Figure 5B–5D, Videos S2, S3, and S4, respectively). However, live mycobacterial phagosomes lost EBP50 faster than other phagosomes (Figure 5C and 5D, Videos S3 and S4). These kinetic studies strongly indicate that live mycobacteria diminish the dwell time of EBP50 on phagosomes resulting in its premature desorption relative to phagosomes with dead mycobacteria or latex beads.

### Coronin-1 Is a Negative Regulator of iNOS Recruitment to Model Phagosomes

**Figure 6:**
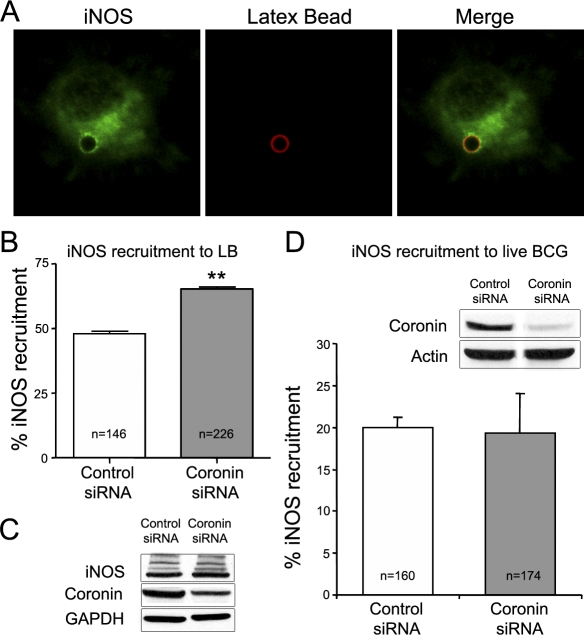
Knockdown of Coronin-1 Increases Localization of iNOS to Model Latex Bead Phagosomes but Not on Live Mycobacteria Phagosomes Macrophages were transfected with either control (scramble) or coronin-1 siRNA, stimulated with IFN-γ and LPS for 16 h the following day, and then incubated with Alexa 568–labeled latex beads or Alexa 568 live mycobacteria for 10 min. After phagocytosis followed by 1-h chase, cells were fixed, stained for iNOS, and examined by confocal microscopy. (A) Localization of iNOS (green) to latex bead phagosomes (red). (B) Quantification of iNOS recruitment to latex bead phagosomes. Bars, mean ± s.e.m. (three independent experiments); *n*, number of phagosomes counted per condition. ^**^ *p* < 0.0001. (C) Western blot of coronin-1 and loading control (GAPDH) after 48 h (includes IFN-γ and LPS stimulation) for (A and B). (D) Quantification of iNOS recruitment to live mycobacteria phagosomes. Bars, mean ± s.e.m. (three independent experiments); *n*, number of phagosomes counted per condition. *p* > 0.05. Inset: western blot of coronin-1 and actin (loading control).

The time-resolved studies with EBP50 localization relative to phagosomes are indicative of the positive role of EBP50 in iNOS localization. However, the transient association of EBP50 with phagosomes suggests that other factors could play a role in maintaining the differences in steady-state distribution of iNOS relative to phagosomes with live mycobacteria versus dead mycobacteria or latex beads. Hence, we investigated another actin-organizing element, coronin-1, known to be recruited to phagosomes with live but not dead mycobacteria [18]. Thus, we hypothesized that recruitment of coronin-1 to live mycobacterial phagosomes could affect iNOS distribution, since our earlier studies showed that actin rearrangements are essential for proper positioning of iNOS [19]. To test this hypothesis, coronin-1 expression was knocked down by siRNA and the distribution of iNOS relative to phagosomes examined. Cells were transfected with either control (scramble) siRNA or coronin-1 siRNA, treated with IFN-γ and LPS for 16 h, and then allowed to phagocytose latex beads. In cells treated with coronin-1 siRNA, there was a significant increase in the recruitment of iNOS to the latex bead phagosome as compared to cells transfected with control siRNA (65.2% ± 0.8% versus 48.0% ± 0.9% *p* < 0.0001) (Figure 6A and 6B). Coronin-1 knockdown expression was confirmed by western blotting (Figure 6C). Next, we tested whether coronin-1 knockdown could rescue iNOS recruitment to live mycobacteria phagosomes. Cells were transfected with either control (scramble) siRNA or coronin-1 siRNA, treated with IFN-γ and LPS for 16 h, and then allowed to phagocytose live mycobacteria. Cells transfected with coronin-1 siRNA showed the same percentage of iNOS localization with live mycobacterial phagosomes relative to cells transfected with control siRNA (Figure 6D). These results show that coronin-1 plays a negative regulatory role in the recruitment of iNOS to model phagosome, but it is not a contributing factor in mycobacteria-induced inhibition of iNOS recruitment to phagosomes.

### Knockdown of EBP50 Promotes Mycobacterial Survival in IFN-γ- and LPS-Activated Macrophages

**Figure 7:**
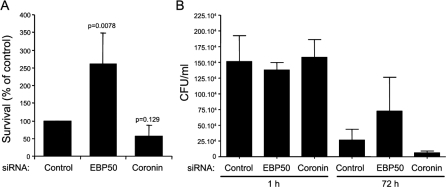
Mycobacterial Killing in IFN-γ- and LPS-Induced Macrophages Is Mediated by EBP50 Murine macrophages were transfected with control (scramble), EBP50, or coronin 1 siRNAs for 48 h, activated with IFN-γ and LPS overnight prior to infection with BCG in the presence of IFN-γ and LPS, and survival determined by CFU counting. The results in (A) are percents relative to control siRNA normalized to 1 h time point or shown as CFU (B). Bars, mean ± s.e.m. (three independent experiments).

If iNOS recruitment to mycobacterial phagosomes occurs via EBP50, then a knockdown of EBP50 should increase survival of mycobacteria in macrophages. In order to test this, RAW 264.7 murine macrophages were transfected with control (scramble), EBP50, or coronin-1 siRNA, activated with IFN-γ and LPS for 16 h, and infected with M. bovis BCG. At indicated time points, cells were lysed and plated for colony forming unit (CFU) determination. The EBP50 knockdown resulted in a significant increase in mycobacterial survival compared to control siRNA (Figure 7). However, no difference in BCG survival was seen in macrophages transfected with coronin-1 siRNA (Figure 7). These results indicate that EBP50 is essential for mycobactericidal activity in LPS- and IFN-γ-treated macrophages.

## Discussion

In this study, we have demonstrated that EBP50 plays a significant role in the localization of iNOS to phagosomes in macrophages. A plausible model emerges with EBP50 linking iNOS and the actin cytoskeleton to phagosomes and explains in part how mycobacteria inhibit iNOS recruitment due to reduced EBP50 dwell time on mycobacterial phagosomes. Our findings, along with previous observations [15], demonstrate that iNOS is not properly localized relative to the phagosomes harboring live M. tuberculosis, and that this localization of iNOS includes an EBP50-dependent mechanism.

M. tuberculosis is an intracellular pathogen that is exceptionally well adapted to ensure its intracellular survival and persistence in macrophages. It has evolved mechanisms to counter multiple and often independently acting bactericidal effectors in phagocytic cells. In this study we have demonstrated that live mycobacteria are able to inhibit localization of iNOS to the phagosome. This finding indicates that the mislocalization of iNOS relative to phagosomes is an active process similar to other phenotypes observed only with live mycobacteria, such as inhibition of phagosome maturation [3,4,18,20]. The mechanism responsible for a decrease in iNOS localization to live mycobacterial phagosomes involves a kinetic change in EBP50 association with mycobacterial phagosomes. Previous observations have demonstrated that the SAL motif at the C-terminal end of iNOS can bind to a PDZ domain of EBP50 in epithelial cells [16]. The SAL motif was further found to be important in the proper apical localization of iNOS and vectorial output of NO in epithelial cells, allowing for maximal efficiency of delivering NO**^.^** to its targets while reducing general cellular toxicity. We found that EBP50 copurifies with iNOS on isolated model phagosomes. Furthermore, EBP50-GFP and iNOS colocalize by immunofluorescence in macrophages. Biochemical and morphological colocalization of iNOS and EBP50 is not coincidental, as functional experiments both with EBP50-GFP and endogenous EBP50 knockdowns have internally consistent effects on iNOS localization. Depletion of endogenous EBP50 by siRNA inhibited iNOS localization to both model and dead mycobacteria phagosomes similar to the effects seen with live mycobacteria. Most importantly, a knockdown of EBP50 interferes with mycobactericidal mechanisms in LPS- and IFN-γ-activated macrophages.

Other pathogens have been reported to interfere with membrane-cytoskeleton linker proteins such as EBP50 and the ERM family of proteins [21,22]. Listeria monocytogenes, another intracellular pathogen, was shown to recruit ERM proteins to form plasma membrane protrusions used for cell-to-cell transmission [22]. These protrusions are dependent on ERM protein interactions with a membrane component and actin, as well as proper phosphorylation of ERM proteins. Another study has identified EBP50 as a molecular target for the Enteropathogenic Escherichia coli effector protein Map [21]. With a motif similar to the PDZ binding motif (SAL) of iNOS, Map uses a TRL sequence to bind to the PDZ1 domain of EBP50. In the case of Map, its binding to EBP50 induces a proteolytic degradation of EBP50, causing a cleavage of the ezrin-binding domain in the Map-bound EBP50, rendering the affected EBP50 unable to associate with its ERM binding partners. With effects on pathophysiology of infections [21], EBP50 modulates the proper localization of its partner ERM proteins [23] in a process that controls its own localization. Taken together, the precedents of pathogen interactions with EBP50 and ERM proteins, the in vitro role of ERM proteins and actin on general phagosome biology [24,25], and the role of EBP50 in iNOS recruitment to phagosomes support the targeting of EBP50 by M. tuberculosis as a process contributing to exclusion of iNOS and mycobacterial survival [21,22,26].

Previously, actin [25,27,28] and the ERM proteins ezrin [25] and moesin [25,27] have been found on model latex bead phagosomes. These reports and our findings indicate that iNOS is most likely recruited by EBP50 and ERM proteins to the vicinity of the phagosomal membrane. An EBP50-iNOS complex is recruited to the phagosome by EBP50, possibly in association with ERM proteins ultimately leading to entrapment of iNOS in an actin network polymerized around the phagosome [25,27,28]. This model of EBP50 playing a role in positioning of iNOS is supported by the observation that endogenous EBP50 levels are increased coordinately with iNOS upon macrophage activation. Macrophage activation-dependent increase in EBP50 levels places this protein into the category of immune response factors.

Coronin-1, which acts as a negative regulator of actin branch polymerization [19,29], still lacks a physiological role explaining its previously reported survival-conferring association with live mycobacterial phagosomes [18], albeit some effects on Ca^2+^ have been recently reported [30]. By being recruited to phagosomes and exerting its anti-actin branching effects [19], coronin-1 may contribute to iNOS localization as demonstrated here in the case of latex bead phagosomes. So why did knockdown of coronin-1 not result in iNOS recruitment to mycobacterial phagosomes? Recently, Deghmane et al. [31] have shown that IFN-γ activation results in disappearance of coronin-1 from live mycobacteria phagosomes. This work explains why a knockdown of coronin-1 did not rescue iNOS recruitment to phagosome containing live mycobacteria. Alternatively, coronin-1 knockdown may not be sufficient to overcome the effect of mycobacteria on iNOS localization, via EBP50. In conclusion, we have described a mechanism endowing mycobacteria to defend themselves against iNOS-dependent microbicidal capabilities of macrophages. Pharmacological approaches aimed at counteracting effects of mycobacteria on EBP50 may therefore lead to increased clearance of *M. tuberculosis.*

## Materials and Methods

### Cell and bacterial cultures, labeling of mycobacteria, and latex beads.

The murine macrophage cell line RAW 264.7 was maintained in DMEM supplemented with 4 mM L-glutamine and 10% fetal bovine serum (FBS). Mycobacterium tuberculosis var. *bovis* BCG was grown in Middlebrook 7H9 broth, and BCG expressing GFP was maintained in 7H9 broth containing 25 μg/ml kanamycin. Mycobacterium was heat-killed for 10 min at 90 °C. Mycobacteria (live and dead) and latex beads (1.0 μm and 3.0 μm) were labeled with Texas Red-succinimidyl ester (0.5 mg/ml), or Alexa 568 succinimidyl ester (5 μg/ml) in PBS for 1 h. Labeled mycobacteria and latex beads were washed three times in PBS, homogenized, and opsonized in DMEM supplemented with 10% FBS.

### Percentage of viability determination.

Viability of bacterial cultures was determined by comparing the OD_600_ counts (where an OD_600_ of 0.1 is equivalent to 1 × 10^7^ cfu/ml) to those obtained by plating for CFU determination. Percentage of viability was 76.05 ± 6.891.

### Plasmid constructs and transfection.

EBP50-GFP was from Dr. S. Lambert (University of Massachusetts Medical School, Massachusetts). Transfection of RAW 264.7 cells was carried out as previously described [4]. Briefly, 5 × 10^6^ cells were resuspended in nucleoporator buffer (Amaxa Biosystems) containing 5 μg of DNA or 1.5-μg siRNA, and nucleoporated according to manufacturer's protocol. Cells were then either plated on cover slips or cultured in flasks 24 h prior to bacterial infections or biochemical experiments.

### Antibodies, cytokines, latex beads, and fluorescent dyes.

Rabbit polyclonal antibody to EBP50 was from Affinity BioReagents. Rabbit polyclonal and mouse monoclonal antibodies against iNOS were from Transduction Laboratories. Mouse monoclonal antibodies against GAPDH and actin were from Abcam. Coronin-1 antibody was from J. Pieters (University of Basel, Switzerland). Murine IFN-γ, LPS, and latex beads were purchased from Sigma-Aldrich. Secondary antibodies conjugated to Alexa 488 and 568 were from Molecular Probes.

### Cytokine treatment of macrophages.

RAW 264.7 macrophages were stimulated with IFN-γ and LPS (200 U/ml and 200 ng/ml). For immunofluorescence experiments, macrophages were seeded onto cover slips in 12-well tissue culture plates at a density of 3.0 × 10^5^ cells per cover slip and exposed to 500 U/ml of IFN-γ and 500 ng/ml of LPS for 16 h prior to infection. For western blotting and purification of latex bead phagosomes, macrophages were incubated with both IFN-γ (500 U/ml) and LPS (500 ng/ml) in their respective flask for 16 h prior to lysis or homogenization.

### Western blotting.

For immunoblotting, cells were washed in PBS and lysed with buffer containing 50 mM Tris HCl (pH 7.4), 150 mM NaCl, 0.25 % deoxycholate, 1 mM EDTA, 1% NP-40, Leupeptin (10 μg/ml), Pepstatin (1 μg/ml), E64 (1.79 μg/ml), TLCK (10 μg/ml), 1 mM activated Na_3_VO_4_, and 1 mM NaF. 50 μg of protein was loaded and separated on a 12% SDS-polyacrylamide gel and transferred to nitrocellulose. Staining was revealed with SuperSignal West Dura chemiluminescent substrate (Pierce). GAPDH and actin were used as a loading control.

### Purification of latex bead phagosomes from macrophages.

Latex bead phagosomal compartments were isolated from latex bead–infected RAW 264.7 macrophages as described previously [32]. Magnetic bead phagosomes were prepared as follows: Macrophages were incubated for 1 h at 37 °C, 5% CO_2_ with magnetic particles (Polysciences) (1/45, v/v) in 150 mM NaCl, 20 mM HEPES (pH 7.4), 6.5 mM glucose, 1 mg/ml BSA. After three washes with cold PBS, cells were lysed in homogenization buffer (HB) (250 mM sucrose, 20 mM Hepes, [pH 7.2], protease inhibitors) by passing through 22-gauge needles connected to a two-syringe apparatus. Post-nuclear-supernatant (PNS) was generated and phagosomes were isolated using a magnetic separator (Polysciences), resuspended, and washed four times with HB. PNS (60 μg) and phagosomes (20 μg) were analyzed by western blot, equal amounts of protein were loaded for untreated and IFN-γ- and LPS-treated cells.

### Immunofluorescence laser scanning confocal microscopy.

RAW 264.7 cells grown on cover slips were fixed with 1% paraformaldehyde followed by membrane permeabilization using 0.2% Saponin or 0.1% Triton X-100. After appropriate antibody incubations, cover slips were mounted using Permafluor Aqueous mounting medium (Immunon). Collection of 1-μm thick optical sections was performed using an Axiovert 200 M microscope with an Axioscope 63× oil objective and LSM 5 Pascal or LSM 510 META systems (Carl Zeiss). Images were cropped using Adobe Photoshop CS2. An average of two phagosomes per cell were counted with an average of 135 cells per condition.

### 4-D confocal microscopy.

Live cell confocal microscopy was performed as previously described [4]. For quantification, maximum intensity projections and mean-intensity projections were used for rendering and quantitative analysis as previously described [17]. Relative fluorescence intensity in cytosol (RFU_c_) was substracted from RFU on phagosome (RFU_p_). Each kinetic was normalized to percentage of maximum RFU (RFU_p_ − RFU_c_).

### Survival assay.

Mycobacteria were homogenized to remove clumps and centrifuged at 800 rpm for 1 min for synchronization of uptake by transfected RAW 264.7 macrophages. Cells were washed three times in PBS and lysed in cold water 1 h and 3 d post-infection. Serial dilutions of cell lysates were plated onto 7H11 plates containing ADC for CFU determination. The results were normalized to 1 h time point and are represented as percent relative to control siRNA. Statistical analysis was done on three independent experiments using Wilcoxon matched pairs test.

## Supporting Information

Video S1 Live 4-D Confocal Microscopy of EBP50-GFP Recruitment to Latex Bead Phagosomes RAW 264.7 cells were transfected with EBP50-GFP (green) and infected with latex beads (red). Frames were taken 80 s apart for a total of 40 min. The video is played at three frames per second. Each frame is a z section of three optical slices collapsed to show an x-y projection of 3.6-μm thickness of the cell.
(77 KB AVI)

Video S2 Live 4-D Confocal Microscopy of EBP50-GFP Recruitment to Dead Mycobacterial Phagosomes RAW 264.7 cells were transfected with EBP50-GFP (green) and infected with heat-killed BCG (red). Frames were taken 80 s apart for a total of 30 min. The video is played at three frames per second. Each frame is a z-section of three optical slices collapsed to show an x-y projection of 3.6-μm thickness of the cell.
(199 KB AVI)

Video S3 Live 4-D Confocal Microscopy of EBP50-GFP Recruitment to Live Mycobacterial Phagosomes RAW 264.7 cells were transfected with EBP50-GFP (green) and infected with live BCG (red). Frames were taken 80 s apart for a total of 30 min. The video is played at three frames per second. Each frame is a z-section of three optical slices collapsed to show an x-y projection of 3.6-μm thickness of the cell.
(150 KB AVI)

Video S4 Click here for additional data file. Live 4-D Confocal Microscopy of EBP-50 Recruitment to Live Mycobacterial Phagasomes
(319 KB AVI)

## Abbreviations

CFU: colony forming unit
ERM: ezrin/radixin/moesin
IFN-γ: interferon-gamma
iNOS: inducible nitric oxide synthase
LPS: lipopolysaccharide
NO ^.^: nitric oxide
RNI: reactive nitrogen intermediate
siRNA: short interfering RNA
